# A Complicated Route from Disorder to Order in Antimony–Tellurium Binary Phase Change Materials

**DOI:** 10.1002/advs.202301021

**Published:** 2023-12-22

**Authors:** Yonghui Zheng, Wenxiong Song, Zhitang Song, Yuanyuan Zhang, Tianjiao Xin, Cheng Liu, Yuan Xue, Sannian Song, Bo Liu, Xiaoling Lin, Vladimir G. Kuznetsov, Ilya I. Tupitsyn, Alexander V. Kolobov, Yan Cheng

**Affiliations:** ^1^ Key Laboratory of Polar Materials and Devices (MOE) Department of Electronics East China Normal University Shanghai 200241 China; ^2^ National Key Laboratory of Materials for Integrated Circuits Shanghai Institute of Microsystem and Information Technology Chinese Academy of Sciences Shanghai 200250 China; ^3^ Suzhou Key Laboratory for Nanophotonic and Nanoelectronic Materials and Its Devices School of Materials Science and Engineering Suzhou University of Science and Technology Suzhou Jiangsu 215009 China; ^4^ The Science and Technology on Reliability Physics and Application of Electronic Component Laboratory China Electronic Product Reliability and Environmental Testing Research Institute Guangzhou Guangdong 511370 China; ^5^ A.F. Ioffe Physico‐Technical Institute RAS St Petersburg 194021 Russia; ^6^ Department of Physics St. Petersburg State University St. Petersburg 199034 Russia; ^7^ Institute of Physics Herzen State Pedagogical University of Russia St Petersburg 191186 Russia

**Keywords:** antimony–tellurium binary materials, atomic structure, phase transition mechanism, phase‐change materials, spherical aberration‐corrected transmission electron microscope

## Abstract

The disorder‐to‐order (crystallization) process in phase‐change materials determines the speed and storage polymorphism of phase‐change memory devices. Only by clarifying the fine‐structure variation can the devices be insightfully designed, and encode and store information. As essential phase‐change parent materials, the crystallized Sb–Te binary system is generally considered to have the cationic/anionic site occupied by Sb/Te atoms. Here, direct atomic identification and simulation demonstrate that the ultrafast crystallization speed of Sb–Te materials is due to the random nature of lattice site occupation by different classes of atoms with the resulting octahedral motifs having high similarity to the amorphous state. It is further proved that after atomic ordering with disordered chemical occupation, chemical ordering takes place, which results in different storage states with different resistance values. These new insights into the complicated route from disorder to order will play an essential role in designing neuromorphic devices with varying polymorphisms.

## Graphical abstract

Direct atomic identification technique demonstrates that the ultrafast speed of Sb–Te materials is due to the disordered nature of lattice occupation by different classes of atoms. After atomic ordering with disordered chemical occupation, is a chemical ordering sequence, which brings different storage states with different resistance values.

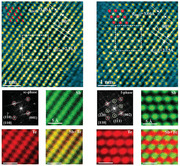

## 1 Introduction

Chalcogenide phase change memory (PCM) has been commercialized for optical recording discs and electrical memory.^[^ ^1^ ^]^ An application example is Intel's Optane series products.^[^ ^2^ ^]^ PCM is also a promising candidate in several state‐of‐the‐art applications, such as neuromorphic computing hardware and nanophotonic devices.^[^ ^3^, ^4^, ^5^, ^6^, ^7^, ^8^ ^]^ All these fascinating applications rely on a reversible transition of a storage medium—i.e., a phase change material—between amorphous (high resistance, low reflectivity) and crystalline (low resistance, high reflectivity) states. The crystallization kinetics is particularly important in storage‐ and computation‐related information applications, because it determines the operating speed of PCM devices and whether the structure‐related polymorphism in neuromorphic devices is sufficiently controllable.^[^ ^9^ ^]^

Generally, the crystallization of phase change materials can be viewed as a disorder‐to‐order transition^[^ ^10^, ^11^, ^12^ ^]^ that undergoes several kinetically favorable metastable states. In the most widely used GeTe–Sb_2_Te_3_ (GST) pseudo‐binary alloys, the amorphous (amor‐) phase first crystallizes into a metastable face‐centered cubic (fcc‐) phase and then into the stable hexagonal (hex‐) phase,^[^ ^13^ ^]^ accompanied by an increase in conductivity. In GST‐based PCM devices, reversible structure switching between the amor‐ and fcc‐phases (≈50 ns) is usually utilized to store information, while the stable hex‐phase is not involved.^[^ ^14^ ^]^ That is because both the amor‐ and fcc‐phases have similar cubic crystal‐like octahedral motifs without obvious vacancy and constituent atoms ordering to ensure a swift transition.^[^ ^15^ ^]^ The metastable cubic phase is not unique to GST ternary alloys, some of the present authors demonstrated that the essential Sb_2_Te_3_ binary alloy also possesses a metastable fcc‐phase.^[^ ^16^ ^]^ Consequently, a faster SET speed was predicted for Sb_2_Te_3_ since its amor‐phase does not contain tetrahedrally coordinated atoms.^[^ ^17^ ^]^ Recently, a fast switching speed was experimentally observed in Sb_2_Te_3_‐based PCRAM devices, for instance, Y_3.7_Sb_40.3_Te_56.0_ (3.2 ns),^[^ ^18^ ^]^ Sc_0.2_Sb_2_Te_3_ (0.7 ns)^[^ ^19^ ^]^ and Er_0.52_Sb_2_Te_3_ (3.2 ns),^[^ ^20^ ^]^ which is inseparable from the parent cubic‐structure's contribution. In the Sb–Te binary system, not only Sb_2_Te_3_ but also Sb‐rich Sb–Te alloys can enable ultrafast speed, evidenced in In_5_Sb_80_Te_15_ film (18 ns),^[^ ^21^ ^]^ and Ta‐doped Sb_2_Te (2 ns).^[^ ^22^ ^]^

In contrast to the cubic arrangement in the Sb_2_Te_3_ alloy, crystalline Sb‐rich Sb–Te alloys are supposed to have a layer trigonal (tri‐) structure, composed of quintuple Sb_2_Te_3_ layers and Sb_2_ bi‐layers.^[^ ^23^ ^]^ On the other hand, Matsunaga et al.^[^ ^24^ ^]^ demonstrated that the crystalline phase in the Ag_3.5_In_3.8_Sb_75.0_Te_17.7_ (AIST) alloy (Sb/Te ratio ≈4.2) possesses the A7 structure ($R\bar{3}m$)—that is not a layered one—where four constituent elements randomly occupy the lattice position. They further proposed a bond–interchange model that only requires a small shift of the Sb atoms within the lattice to achieve a high‐speed transition.^[^ ^25^ ^]^ Although the Sb–Te binary system shows the ability to achieve ultrahigh‐speed phase switching, until now no universal rapid phase transition theory exists. Some literature sources have analyzed the corresponding phase transition behavior from the perspectives of conductivity,^[^ ^26^ ^]^ X‐ray diffraction (XRD),^[^ ^24^ ^]^ etc., but few attempts have been made to directly elucidate the detailed crystallization process, pathway, and structure change on an atomic scale. Considering the similarity of constituent elements in the Sb–Te system, it's reasonable to conclude that if we can understand the complicated route from disorder to order in Sb–Te binary, it would be helpful to clarify the rapid phase transformation observed in these materials, both stoichiometric Sb_2_Te_3_ and Sb‐rich alloys.

Here, with state‐of‐the‐art direct atomic identification and in situ biasing inside spherical aberration (Cs)‐corrected transmission electron microscope (TEM), we found that the initial step of the crystallization of Sb–Te alloys is atomic ordering with chemical disorder, and then chemical ordering takes place, concomitant with a decrease in the system energy and resistance. The disordered occupation of lattice sites was present throughout the Sb–Te binary system during crystallization, whose octahedral structural motifs are very similar to those in the amor‐phase, ensuring ultrahigh phase switching speed. As the anions and cations gradually spatially separate, the structure can be fine‐tuned, accompanied by a further reduction in electrical resistance. We propose that this cognition will provide useful knowledge for understanding rapid crystallization mechanisms, developing ultrahigh‐speed phase change materials, and designing structurally stable and controllable neuromorphic devices.

## 2 Results and Discussion

The crystallization process of amor‐Sb_2_Te_3_ was first investigated using ab initio molecular dynamics (AIMD) simulations, the results being shown in Supporting Information (Figure S1–S3, Supporting Information). The details of the simulations are provided in methods. One can see at the initial crystalline state, ordered atomic planes started to form from the random structure. Inspecting single atomic planes, one can clearly see that cubic (square when one considers planes) lattice sites are randomly occupied by Sb and Te atoms. While the relatively small simulation cell size (for more realistic crystallization studies cells with the number of atoms on the order of 500 should be used^[^ ^27^ ^]^) may be prone to artifacts affecting the processing speed, the obtained results from two simulated supercells of different sizes suggest the formation of a simple cubic (sc‐) structure at the initial stage of crystallization, that is to say, there is no separation of anions and cations during the atomic ordering process (AOP). This observation served as a guideline for a detained experimental study of the crystallization process of amor‐Sb_2_Te_3_.

**Figure 1:**
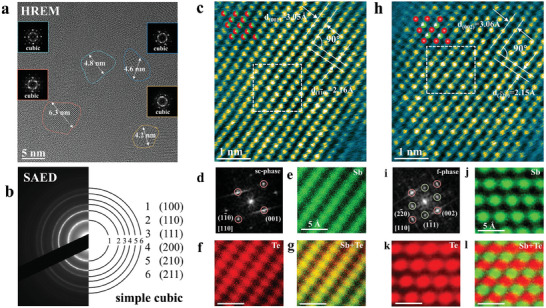
The microstructure of an as‐deposited Sb_2_Te_3_ film. a) High‐resolution electron microscopy (HREM) imaging indicates that the grain size was ≈5 nm in the polycrystalline film. b) One can index the corresponding selected area electron diffraction (SAED) pattern of panel a as a simple cubic (sc‐) phase. c) High‐angle annular dark field (HAADF) images of sc‐phase projected along the [110] orientation. d) The fast‐Fourier transform (FFT) patterns of panel c. e–g) Atomic energy‐dispersive X‐ray spectroscopy (EDS) mappings of elements Sb and Te as well as the overlap, respectively; from the white dotted box in panel c. h) HAADF images of face‐centered cubic (fcc‐) phase projected along the [110] orientation. i) The corresponding FFT of panel h. j–l) The atomic EDS mappings of elements Sb and Te as well as the overlap, respectively; from the white dotted box in panel h.

Experimentally, the as‐deposited Sb_2_Te_3_ film after deposition manifests polycrystalline morphology. Here, we prepared a Sb_2_Te_3_ film by co‐sputtering Sb and Te targets at room temperature to investigate its initial crystalline structure. **Figure** **1**a shows a high‐resolution electron microscopy (HREM) image of an as‐deposited Sb_2_Te_3_ film, indicating randomly distributed cubic nano‐grains (≈5 nm in size). The corresponding continuous selected area electron diffraction (SAED) rings in Figure 1b also indicate polycrystallinity. By comparing the square radius of the diffraction rings in reciprocal space, the sequential ratio from the inside to the outside fits well with the selection rules of the sc‐phase, space group $Pm\bar{3}m$ ^[^ ^28^ ^]^ (Table S1, Supporting Information). The sc‐structure indicates that the constituent atoms are randomly located on the cubic lattice sites, consistent with the simulation results, where the smallest repeatable structural unit (a = 3.05 Å) is halved in size compared with the fcc‐phase (a = 6.10 Å).^[^ ^17^ ^]^ Atomic‐scale Cs‐TEM was used to further evaluate the details of the atomic arrangement. Figure 1c,d shows a scanning transmission electron microscopy high angle annular dark field (STEM‐HAADF) image of a nanocrystalline sc‐phase grain and its corresponding fast‐Fourier transform (FFT) pattern projected along the [110] direction. The energy dispersive X‐ray spectroscopy (EDS) mappings and the overlap of Sb and Te at the atomic scale in Figure 1e–g clearly demonstrate that the Sb and Te atoms are randomly distributed on the sc‐phase lattice sites. To contrast with the fcc‐phase, the microstructure of a [110] orientated fcc‐grain was also investigated as shown in Figure 1h–l, prepared by an Sb_2_Te_3_ alloy target. The main microstructure differences between the two cubic phases are as follows. First, the HAADF image of sc‐Sb_2_Te_3_ in Figure 1c is characterized by a uniform contrast in the atomic columns, whereas the HAADF image of fcc‐Sb_2_Te_3_ with the same orientation as in Figure 1h clearly exhibits a periodic variation in contrast. In accordance with the incoherent scattering intrinsic of HAADF,^[^ ^28^, ^29^ ^]^ the contrast is roughly proportional to Z^2^, where Z is the atomic number. The atomic numbers of Sb and Te are 51 and 52, respectively; hence they would have a similar contrast weight in HAADF images. Thus, the uniform contrast in the sc‐phase (Figure 1c) demonstrates that the average atomic number, contributed by the sum of the occupancy of Sb and Te atoms, is nearly the same in each atomic column. On the other hand, the periodicity variation contrast in the fcc‐phase (Figure 1h) demonstrates that the average atomic number in the dark atomic columns is smaller, which is ascribed to the existence of a cationic vacancy in the fcc‐phase. This is also fully consistent with the simulated HAADF images of the two phases in Figure S4 (Supporting Information). Additionally, the distribution of the main spots (pink circles) in FFT patterns obtained from the two phases is rather similar in sc‐Sb_2_Te_3_ (Figure 1d) and fcc‐Sb_2_Te_3_ (Figure 1i) but the fcc‐phase exhibited additional (111) diffraction spots (green circles), attributable to the periodical atomic arrangement variation as observed in the HAADF image. The same distribution of the diffraction spots was also depicted in the simulated FFT images (Figure S5, Supporting Information). Finally, unambiguous experimental evidence comes from the direct atomic resolution EDS mapping, which shows that the signal intensity of the Sb and Te atoms in the sc‐phase (Figure 1e–g) was basically the same in each atomic column; whereas the two elements alternatively and exclusively occupied the (111) lattice plane of the fcc‐phase (Figure 1j–l), exhibiting periodical signal intensity. Therefore, the initial crystallized structure in the Sb_2_Te_3_ film is the sc‐phase with randomly distributed Sb and Te atoms on the cubic lattice sites as suggested by our ab initio simulations.

The randomly distributed constituent atoms have been previously discussed in GST alloy; for instance, a body‐centered cubic structure forms under hydrostatic compression before amorphization^[^ ^30^, ^31^ ^]^ as confirmed by XRD experiments; a random distribution of Ge and Sb atoms and a small fraction (of order 3%) of antisite Te defects on the cation sublattice in cubic phase^[^ ^32^ ^]^ or an sc‐phase^[^ ^33^ ^]^ as suggested by theoretical simulations but without experimental evidence. Compared with GST ternary alloy, Sb_2_Te_3_ is a binary phase change material without Ge atoms, but from the theory and experiment results we have found that sc‐phase can also be formed in it during the crystallization process. Such a random atomic arrangement in the crystallized state is similar to the structure observed in high‐entropy alloys,^[^ ^34^ ^]^ indicating that a large configurational entropy in the free energy aids the crystallization process from the perspective of reaction thermodynamic as reported in GST alloy.^[^ ^27^ ^]^ The fact that the lattice constant of the fcc‐phase is twice the value of that in the sc‐phase strongly suggests that the fcc‐phase forms from the sc‐phase through a chemical ordering process (COP). This assumption was verified by direct observation of the phase transition from sc‐ to fcc‐phase at elevated temperature in TEM (Figures S6 and S7, Supporting Information). In the sc‐phase, the constituent atoms remained disordered, making them more similar to the amor phase.

It was shown that the switching speed of doped‐Sb_2_Te_3_ devices is very fast, for instance, the switching time is only 700 ps in Sc_0.2_Sb_2_Te_3_ alloy.^[^ ^19^ ^]^ We also noticed that elemental Sb‐based devices, whose crystallization process only involves AOP,^[^ ^35^ ^]^ are characterized by an extremely fast crystallization speed within 360 ps. Consequently, if we can control the crystallization process of Sb_2_Te_3_ alloy between the amor‐phase and sc‐phase, which just undergoes the AOP, then the needed duration time is possible on the picosecond time scale. Besides, the preparation method could affect the phase structure after deposition. Co‐sputtering using Sb and Te single targets favored the formation of the sc‐phase, whereas the use of an alloy target tended to produce the fcc‐phase (Figure S8, Tables S1 and S2, Supporting Information), which may be ascribed to the distribution difference of the Sb and Te atoms. The sputtering process is a nonequilibrium process,^[^ ^36^ ^]^ which possesses homopolar bonds and non‐chemically ordered configurations. Compared with single‐target sputtering, the two elements are not sputtered from the same target during the co‐sputtering process, which makes it easier to generate atomic fluxes and the resulting random covalent network. Thus, the distribution of the atoms is more stochastic during a co‐sputtering process. Further, doping with appropriate elements is also an effective method to improve the degree of stochastic distribution to retain the sc‐structure in the crystallized films (Figure S9, Supporting Information) and even in the practical electrical devices (Figure S10, Supporting Information). Thus, the reported rapid switching speed in Sb_2_Te_3_‐doped materials^[^ ^18^, ^19^, ^20^ ^]^ seems to be related to incomplete ordering during crystallization, which only completes AOP. Subsequent COP relates to the atomic migration process that requires a longer time.

**Figure 2:**
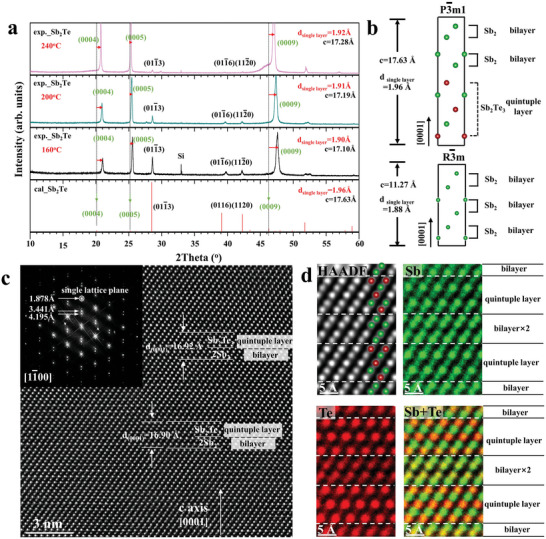
Atomic arrangement variation in an annealed Sb_2_Te film. a) X‐ray diffraction patterns of a Sb_2_Te film annealed at various temperatures (160, 200, and 240 °C). Red arrows denote the mismatch between experimental patterns and theoretical patterns of standard Sb_2_Te, demonstrating that the structure gradually transformed into standard Sb_2_Te with increasing temperature. b) Atomic modeling of standard Sb_2_Te and Sb structures indicate that Sb had a short single‐layer distance. c) HREM image of a crystallized Sb_2_Te film, annealed at 160 °C and projected along the $\left[1\bar{1}00\right]$ orientation; the inset is the corresponding FFT pattern, the single layer distance of which was only 1.878 Å and smaller than the standard value of Sb_2_Te. d) HAADF image of crystallized Sb_2_Te, and the corresponding atomic EDS mappings of elements Sb and Te as well as the overlap; indicating a thorough mixing of elements during the initial crystallization of Sb_2_Te.

The AOP and COP were also observed during the crystallization process of SbTe alloy with an increased concentration of Sb (Figures S11 and S12, Supporting Information). When the Sb concentration in the Sb–Te system is higher than 50%, the rock‐salt cubic structure in a crystalline state cannot be maintained because the presence of vacancies in the Te sites destroys the cubic framework.^[^ ^37^ ^]^ It has been reported that the structure of Sb‐rich Sb–Te materials exhibits tri‐/hex‐crystal phase with a layered structure, but also exhibits ultrafast phase transition ability.^[^ ^38^, ^39^ ^]^ This is a bit confusing because a layered structure takes time to form. To investigate the structural changes during crystallization, we studied the atomic structure arrangement of Sb‐rich Sb–Te alloy (taking Sb_2_Te and Sb_3_Te as examples), which might help establish a general theory for elucidating the fast transition mechanism in Sb–Te alloys. **Figure** **2**a shows XRD patterns of Sb_2_Te films at various temperatures (160, 200, and 240 °C). Because of the texture phenomenon, the (0004), (0005), and (0009) characteristic peaks were distinct. However, these peaks at low temperatures, such as 160 °C, are located at higher angle positions (*d_exp single layer_* = 1.90 Å) than the theoretical peaks in Sb_2_Te (*d_theory single layer_* = 1.96 Å), i.e., exhibiting a smaller *c* axis. By comparing with the theoretical value in the $P\bar{3}m1$ structure (hex‐phase) and the $R\bar{3}m$ structure (A7‐phase) shown in Figure 2b, the measured atomic spacing was closer to the A7 structure (*d_theory single layer_* = 1.88 Å). As the temperature increased, the peak positions gradually shifted to lower angles, corresponding to the increasing *c*‐axis. A similar phenomenon has been observed in Sb‐rich Sb–Te alloy^[^ ^24^, ^40^, ^41^ ^]^ in XRD patterns, which was attributed to vacancies dissolving in the Sb layer (albeit without direct experimental evidence).^[^ ^42^ ^]^ In fact, the calculated *R*‐weighted factor did not show substantial differences when exchanging Sb (Z = 51) with Te (Z = 52) in the atomic model, indicating that distinguishing the two elements through XRD measurements is difficult.^[^ ^43^ ^]^ Direct atomic recognition technology is an effective method to solve this issue. Figure 2c is an atomic‐scale HREM image taken from a $\left[1\bar{1}00\right]$‐oriented grain in a crystallized Sb_2_Te film (annealed at 160 °C), which exhibited a partially layer‐ordered structure. From top to bottom, the repetition period along the [0001] direction consisted of a quintuple layer and bilayer, which might correspond to the theoretical Sb_2_Te_3_ and Sb_2_ blocks, respectively. The measured average *c*‐axis (≈16.90 Å) was small, consistent with the XRD results. Figure 2d shows a typical enlarged HAADF image, and the corresponding direct atomic recognition mappings of Sb and Te as well as their overlap. Clearly, the bilayer block was not exclusively occupied by Sb atoms, but also contained a large proportion of the Te atoms, i.e., some features of the random distribution of constituent atoms in the A7 structure were preserved. Similarly in the quintuple block, a certain fraction of Sb atoms was in the theoretically predicted Te position. Nevertheless, the signal intensity of the two elements still exhibited periodical contrast variation, hence the bilayer and quintuple layer were also formed. However due to the mixing of anions and cations, the *c*‐axis was shorter, and the structure was similar to the A7 phase. If the mixing degree is higher, then the A7 structure would be expected. In fact, the presence of the A7 structure was observed in an initially crystallized Sb_2_Te film (Figure S13, Supporting Information), which contains twisted octahedral motifs. Such distorted octahedral motifs were also observed in an amor‐Sb_2_Te film through angstrom‐beam electron diffraction patterns (Figure S14, Supporting Information). Thus, it is reasonable to speculate that the crystallization pathway of Sb_2_Te alloy is similar to Sb_2_Te_3_, which also starts from AOP in the initial crystallization stage, showing the A7 structure. As the crystallization continues, the chemical disorder‐to‐order process occurs and forms ordered atomic arrangements along the [0001] direction in a manner that facilitates the gradual expansion of the *c*‐axis. We also noticed a recent theoretical simulation result that found that the crystallization process of Sb_2_Te supercell (810 atoms) is a disorder‐to‐order process, accompanied by a gradual change in energy and optical properties.^[^ ^44^ ^]^ Element doping can effectively delay COP and maintain A7 structure after crystallization (Figure S15, Supporting Information), demonstrating potential access to this metastable phase in practical devices.^[^ ^22^ ^]^ It is also very interesting to note that a similar process, i.e., the establishment of a long‐range order (the formation of layers) while preserving a short‐range (chemical) disorder was observed during the crystallization of Cr_2_Ge_2_Te_6_,^[^ ^45^ ^]^ suggesting that a complex disorder to order phenomenon is general in layered chalcogenides.

**Figure 3:**
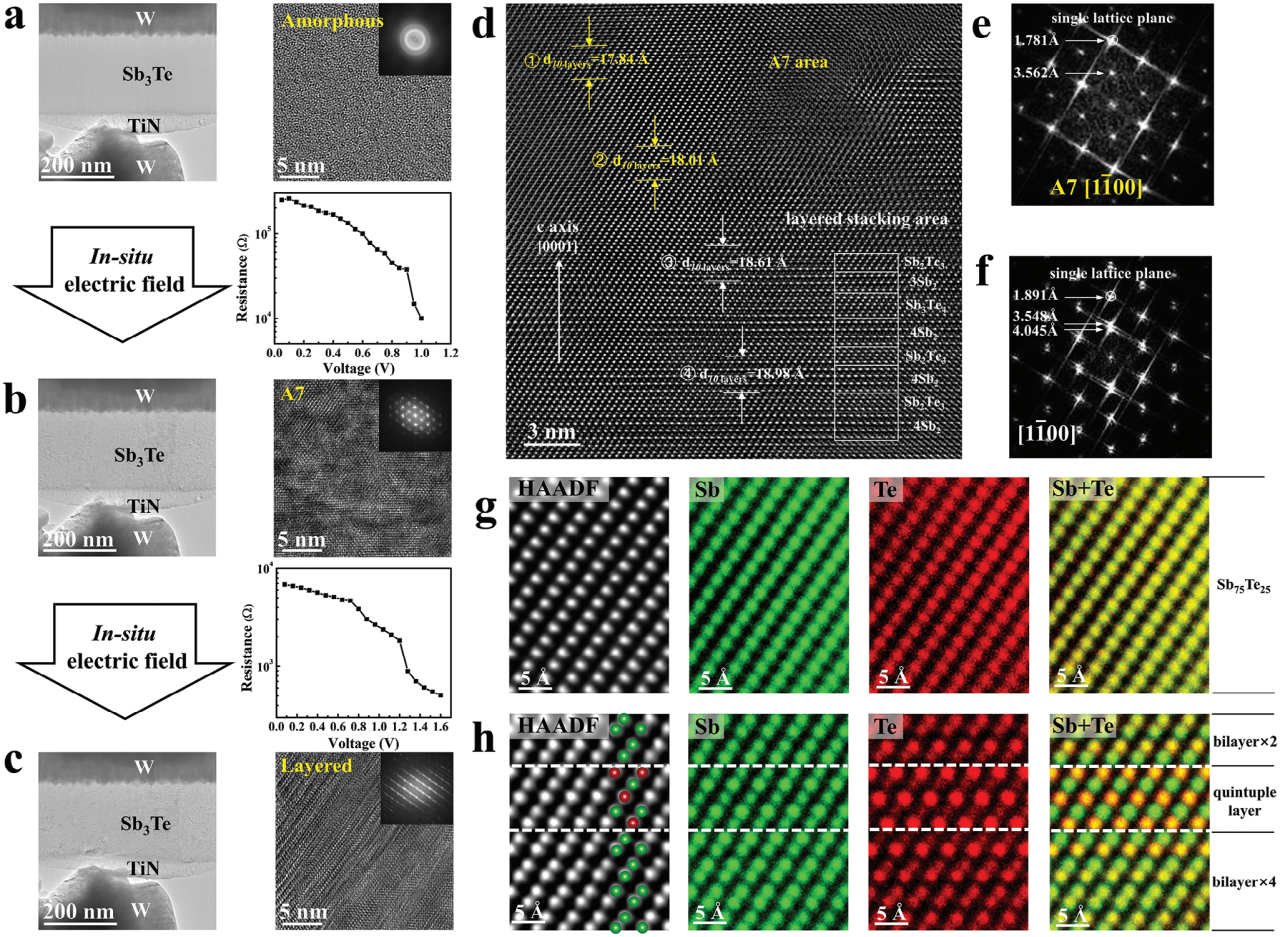
The crystallization evolution in Sb_3_Te. a) TEM image of a W/Sb_3_Te/TiN/W nanodevice, HREM image, and its corresponding FFT pattern identified amorphous state prior to electrical operation. Under in situ electric field, b) the nanodevice crystallizes into A7 state with a decrease of resistance. Another electric field stimulation drives c) the nanodevice into a layered structure with a further decrease of resistance. d) HREM images of a crystallized Sb_3_Te film projected along the $\left[1\bar{1}00\right]$ orientation indicates the transition from A7 to $P\bar{3}m1$ structure from top to bottom, evidenced by the obvious gradual periodic modulation structure, the increased distance of every 10 layers, and splitting spots in the e, f) FFT patterns. g) HAADF image of the A7 area, in addition to corresponding atomic EDS mappings of elements Sb and Te as well as the overlap. The two elements occupy random positions. h) HAADF image of an obvious periodic modulation area, in addition to corresponding atomic EDS mappings of elements Sb and Te as well as the overlap. The two elements still occupy every lattice position but tend to form Sb_2_Te_3_ and Sb_2_ blocks, indicative of chemical ordering.

With a further increase in the Sb content in the Sb–Te system, we observed a gradual peak shift in the XRD patterns in the crystalline state, which became increasingly close to that of the A7 phase (Figure S16, Supporting Information). Hence, Sb_3_Te with a higher Sb content was chosen to investigate the crystallization sequence. First, the structure transition in an electrode/Sb_3_Te/electrode nanodevice (Figure S17, Supporting Information) under an electric field is studied inside Cs‐TEM. **Figure** **3**a shows the W/Sb_3_Te/TiN/W nanodevice before the electrical operation, in which the HREM image and its corresponding FFT pattern show the identified amorphous state. When an in situ electric field is applied to the nanodevice, Figure 3b clearly shows that Sb_3_Te film crystallizes into the A7 structure first, accompanied by a decrease in resistance of more than 1 order of magnitude. With further application of the electric field, as shown in Figure 3c, the A7 phase transforms into a layered structure (the structure difference between the two phases was discussed in Figure 3d–h), accompanied by a further slight decrease in resistance (Movie S1, Supporting Information records the formation process of the layered structure). This in situ electric field experiment clearly shows that the crystallization sequence of Sb_3_Te alloy is from an amorphous to the A7 phase and then to the layered tri‐/hex‐structure. The atomic arrangement of the phase transition area containing the A7 and layered structures is also demonstrated in Figure 3d. From top to bottom, the distance of every ten atomic layers along the *c*‐axis gradually increased from 17.84 to 18.98 Å. In the upper region, no obvious layered structure was detected; whereas a bilayer, a quintuple layer, and even seven layers (denoted by white rectangles) were evident in the bottom. Compared to the A7 phase, the periodical modulation feature in the layered structure was also reflected in the splitting of the diffraction spots (Figure 3e,f). In the upper A7 region, atomic EDS results (Figure 3g) demonstrate that the Sb and Te atoms were randomly distributed at each lattice site. However, the layered area (Figure 3h) tends to be built sequentially from modulated Sb_2_ and Sb_2_Te_3_ blocks, whose atomic columns are co‐occupied by partially ordered Sb and Te atoms. Although the COP was a bit slow from top to bottom, it still can be seen that the distribution of the two elements gradually changed from random to spatially separated/ordered. In short, the above results unambiguously demonstrate that the crystallization process undergoes sequentially the AOP and COP stages, accompanied by a continuous reduction in resistance. This phenomenon also indicates that the polymorphisms in neuromorphic devices are structurally related to the AOP and COP.

**Figure 4:**
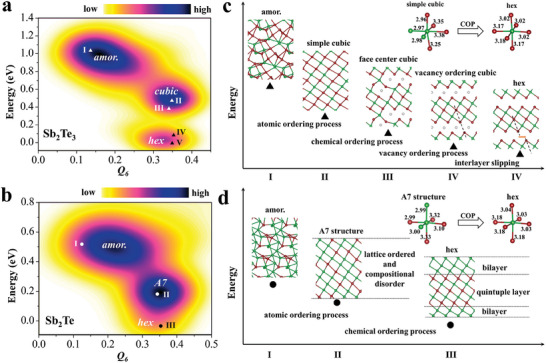
Rapid transition mechanism of an Sb–Te alloy. The total energy (*E*) per unit cell of the minimum versus the Steinhardt order parameter *Q_6_* (degree *l* = 6) for a) Sb_2_Te_3_ and b) Sb_2_Te. The energy *E* is with respect to the global minimum (most‐stable hex‐phase). Snapshots for the phase transition process in c) Sb_2_Te_3_ and d) Sb_2_Te.

To map the crystallization pathway in Sb–Te alloys, we sampled the phase space to obtain the potential energy surface (PES) of Sb_2_Te_3_ and Sb_2_Te systems, in which the stochastic surface walking global optimization^[^ ^46^ ^]^ was used to investigate a variety of possible local structure motifs. **Figure** **4**a,b shows the total energy (*E*) per unit cell of the minimum versus a common structure fingerprint (i.e., the Steinhardt order parameter^[^ ^47^ ^]^ with degree *l* = 6, *Q*_6_) for Sb_2_Te_3_ and Sb_2_Te, respectively. *Q_6_* is the order parameter of the local environment, which is substantially different between crystalline and amorphous states. By carefully examining all structures in the global PES, we identified three major phase zones (in Figure 4a,b) in accordance with the energy differences. In both alloys, the high‐energy area (which spanned a large domain) pertained to the amorphous phase. The intermediate‐energy area was occupied by metastable phases, such as the sc‐phase in Sb_2_Te_3_ and the A7 structure in Sb_2_Te. The lowest‐energy area was the thermodynamically stable hex‐phase. The PES thus describes the optimal crystallization pathway: 1) the amor‐to‐sc/A7 structural transition is a fast diffusionless AOP, driven by thermodynamics; subsequently, 2) the sc‐to‐fcc‐phase (or A7‐to‐hex‐phase) transition is a slowly diffusing COP, until the chemically stable phase is reached.

The entire process of atomic arrangement from disorder to order is demonstrated in Figure 4c,d, with the development of the octahedral motif and reduction of the system energy (the coordinates of which are included in Supporting Information). In the amor‐phase, Sb and Te atoms are randomly distributed with the highest possible energy. As crystallization begins, AOP occurs to form a metastable phase, which is the sc‐lattice for Sb_2_Te_3_ with octahedral motif and the A7‐structure in Sb‐rich Sb–Te system with twisting octahedral motif. AOP is essentially a diffusion‐free process, which retains many Sb–Sb and Te–Te homopolar bonds in the resulting octahedral motif (insets in Figure 4c,d), and thus has an ultrafast crystallization speed. Since AOP corresponds to an abrupt change from a disordered amor‐state to an atomically ordered state at the nanoscale scale, it shows the characteristics of first‐order phase transition.^[^ ^48^ ^]^ Subsequently, Sb and Te atoms gradually separate to the cationic and anionic sub‐lattices, respectively, and form stable bilayer and quintuple layer blocks, termed COP. The above multistep crystallization process was also previously discussed in GST alloy from the perspective of Ostwald's step rule, which notes the prevalence of local octahedral structural units, and energy minimization leads to a dramatic reduction in the number of “wrong bonds” to achieve disorder to order crystallization process.^[^ ^49^ ^]^ In the Sb_2_Te_3_ system, the fcc‐phase possesses ≈33% vacancies at cationic sites after COP. Experimentally, we indeed observed the vacancy clustering and ordering in the (111) lattice plane (Figure S18, Supporting Information), accompanied by a further decrease in the Sb_2_Te_3_ system energy, forming a vacancy‐ordered structure.^[^ ^17^ ^]^ When, due to the formation of vacancy ordering layers, the fcc‐phase cannot sustain, a slipping process—similar to martensitic shear—takes place in a manner that forms the thermodynamically stable hex‐phase.^[^ ^50^ ^]^ In the Sb‐rich Sb–Te system, the stable hex‐phase consisting of bilayer and quintuple layer blocks forms after COP, which is a time‐consuming diffusion process driven by a further decrease of energy. Thus, the COP corresponds to the stepwise chemical ordering process of Sb and Te atoms, showing the characteristics of second‐order phase transition as observed in CuZn metal alloy.^[^ ^51^ ^]^

## 3 Conclusion

In summary, our ab initio simulations suggested and subsequent direct atomic identification unambiguously demonstrated that the crystallization pathway in Sb–Te binary alloys starts with AOP, where the constituent atoms randomly occupy lattice points in the initial crystallized metastable phase. AOP is followed by COP, in which the Sb and Te atoms spatially separate and form cation and anion sublattices, respectively. The entire crystallization process is thermodynamics and kinetics driven by a decrease in the system energy, accompanied by a decrease of resistance. Since the local order in the crystal phase formed as a result of AOP has the highest similarity to the local order of amor‐phase, this process is very fast and can be utilized in practical PCM devices if we can control the phase transition between amor‐phase and compositionally disordered metastable structures. This is possible by element doping in the Sb–Te system, which can delay COP, in other words, promote the thermal stability of the mixed metastable structures. Furthermore, considering that different structures correspond to different electrical properties and, consequently polymorphic states are necessary for the development of neuromorphic computing systems, this work provides important inspiration for the design of artificial synapses and neurons based on phase change materials. Clarifying the mechanism of the crystallization pathway in Sb–Te binary alloys may also have directive significance in more complex phase transition systems.

## 4 Experimental Section

### Sample Preparation

Different compositions of Sb–Te films were deposited by magnetron co‐sputtering using Sb and Te targets at room temperature, the Sb/Te ratio in the grown films was determined through the power applied to the targets and verified by energy‐dispersive X‐ray spectroscopy. The base pressure of the vacuum system was about 2.1 × 10^−4^ Pa, the Ar flow rate was 80 sccm, and the corresponding sputtering pressure was about 0.31 Pa. Films were deposited on Si substrates for XRD spectroscopy, and films deposited on carbon‐coated TEM grid for TEM analyses. After deposition, the films underwent an annealing process at different temperatures in rapid thermal processing in a nitrogen atmosphere. For the in situ TEM experiment, W, Sb_3_Te, and TiN were sequentially deposited on the Si substrate. Then, the W/Sb_3_Te/TiN nano‐pillar device was fabricated on a Cu grid by using the standard focus ion beam (FIB) technique in the Helios G4 instrument.

### Microstructure Characterization

The HREM and STEM analyses were carried out on a JEM Grand ARM300F microscope with double spherical aberration (Cs) correctors. HAADF images in the STEM mode use the probe convergence semi‐angle of ≈24 mrad. The STEM‐HAADF images were taken by an annular dark field image detector with the inner semi‐angle larger than 64 mrad. Two windowless EDS detectors, each of which has an active area of 100 mm^2^, were equipped on the JEM Grand ARM300F microscope, which were very close to the specimen with a high solid angle (1.7 sr). The energy resolution of the detector was 128 eV. During the EDS measurement, the excitation lines that were used were the Lα line for Sb (Lα = 3.604 keV) and Lα line for Te (Lα = 3.769 keV), respectively. The energy difference between the two lines was 165 eV, which was larger than the energy resolution of the detector, therefore the EDS measurement can effectively distinguish the distribution of the two elements. By using the EDS detectors, the average concentrations of Sb and Te elements for Sb_2_Te_3_ were determined to be 35.1 and 64.9 at%, for Sb_2_Te were determined to be 65.0 and 35.0 at%, for Sb_3_Te were determined to be 74.7 and 25.3 at%. The W/Sb_3_Te/TiN nano‐pillar device was mounted onto an X‐nano TEM holder developed at the Center for X‐Mechanics. During the in situ crystallization experiment, the W tip is movable in 3D to contact the top area of a W/Sb_3_Te/TiN nano‐pillar device to form an electrical circuit among W/Sb_3_Te/TiN nano‐pillar device, the W tip and the external electrical facility (Keithley 2450). After the contact formation, a gradually increased DC voltage was applied in the circuit to stimulate the crystallization evolution in the Sb_3_Te alloy. Simultaneously, the current value at different voltages was measured and recalculated into the resistance.

### Simulation

Ab initio molecular dynamics (AIMD) simulations were performed using density functional theory (DFT) within the generalized gradient approximation (GGA) with the PBE exchange‐correlation functional^[^ ^52^ ^]^ and the plane wave basis set as implemented in the pseudopotential‐based CASTEP code.^[^ ^53^, ^54^ ^]^ To describe the electron–ionic interactions, the Vanderbilt ultrasoft pseudopotentials (USP)^[^ ^55^ ^]^ and a cutoff energy E_cut‐off_ = 230 eV were chosen. Taking into account the relatively large size of the supercells (a = b = 13.005 Å; c = 30.559 Å; α = β = 90°; γ = 120° for 135 atoms. a = 16.48 Å, b = 20.02 Å, c = 19.92 Å; α = 97°, β = 85°; γ = 86° for 200 atoms); and consequently the small volume of the Brillouin zone (BZ) used for AIMD simulations, only G‐point was used when integrating over the BZ.

The amorphous phase was generated using a standard melt–quench procedure.^[^ ^56^ ^]^ To account for thermal expansion and the lower density of the amorphous phase, experimental lattice constants increased by 2% were used. This crystalline configuration was heated to 3000 K and held at this temperature to randomize the atomic distribution. The simulations were carried out using the NVT ensemble with a Langevin thermostat (to maintain ergodicity) for an overall time of 15 ps with a time step = 4 fs. Subsequently, the molten structure was cooled down to 1200 K and was allowed to equilibrate for another 15 ps, after which it was quenched to 300 K at a rate of 15 K ps^−1^. In the obtained amor‐phase, Sb and Te atoms were randomly distributed in 3D space. The thus obtained random atomic structure was annealed for over 200 ps at 850 K (somewhat below the melting point) to simulate the crystallization process (using the NVT ensemble with a Langevin thermostat).

The stochastic surface walking (SSW) method in combination with DFT calculations was utilized to sample the phase space of Sb_2_Te_3_ and Sb_2_Te exhaustively. The SSW algorithm^[^ ^57^ ^]^ has an automated climbing mechanism to manipulate a structure configuration from a minimum to a high‐energy configuration along one random mode direction.

## Conflict of Interest

The authors declare no conflict of interest.

## Supporting information

Supporting Information

Supplemental Movie 1

## Data Availability

The data that support the findings of this study are available from the corresponding author upon reasonable request.
