# Supplementary material for: A Complicated Route from Disorder to Order in Antimony–Tellurium Binary Phase Change Materials
Source: Adv Sci (Weinh). 2023 Dec 22;11(9):2301021. doi: 10.1002/advs.202301021 (PMC10916584; doi:10.1002/advs.202301021)
Supplement: Supplementary file 1 — Supporting Information [file ADVS-11-2301021-s001.pdf]

ADVANCED SCIENCE
Open Access

Supporting Information

for *Adv. Sci.*, DOI 10.1002/advs.202301021

A Complicated Route from Disorder to Order in Antimony–Tellurium Binary Phase Change Materials

Yonghui Zheng, Wenxiong Song, Zhitang Song, Yuanyuan Zhang, Tianjiao Xin, Cheng Liu, Yuan Xue, Sannian Song, Bo Liu, Xiaoling Lin, Vladimir G. Kuznetsov, Ilya I. Tupitsyn, Alexander V. Kolobov* and Yan Cheng**

Supporting Information

A complicated route from disorder to order in antimony–tellurium binary phase change materials

Yonghui Zheng, Wenxiong Song, Zhitang Song, Yuanyuan Zhang, Tianjiao Xin, Cheng Liu, Yuan Xue, Sannian Song, Bo Liu, Xiaoling Lin, Vladimir G. Kuznetsov, Ilya I. Tupitsyn, Alexander V. Kolobov, Yan Cheng

Table of contents

- **Simulated crystallization process in Sb_2Te_3**
- **Figures S1-S8**
- **Table S1-S2**
- **Figure S9-S17**
- **Video S1**
- **Figure S18**
- **Coordinates of Sb_2Te_3 systems**
- **Coordinates of Sb_2Te systems**
- **References**

Simulated crystallization process in Sb_2Te_3

Figure S1a shows the evolution of amorpous (amor-) Sb_2Te_3 at various periods of time at 850 K. After an annealing time as brief as 30 ps, longer correlations started to establish, manifested as the formation of ordered atomic layers (Figure S1a). As the time increased to 92 ps, the ordered crystal structure was basically formed. After 146 ps, the crystalline structure was fully established; further annealing to 202 ps did not lead to a substantial structure change. Figure S1b shows the atomic arrangement in a single atomic plane (denoted with an arrow in

Figure S1a) at different periods of time. Ordered cubic (square) atomic arrangements were already identifiable at 30 ps with lattice sites randomly occupied by Sb and Te atoms, indicative of an ultrafast phase change speed.

Ordering is also clearly visible from the radial distributin functions (Figure S2). While the staring amorphous phase only has one first-nearest-neighbor peak, in the annealed samples peaks representing higher coordination shells characteristic of the ordered phase emerge.

The random square-like distribution of atoms must be similar to a disordered amorphous phase. Indeed, in amor-Sb_2Te_3, Caravati *et al.* found that the bond angles of Sb-Te are mostly distributed at ca. 90° and 170°[1]; and Elliot *et al.* further reported that hypervalent interactions, manifested as near-linear chains with alternating long and short bonds, is the basic geometric structural pattern[2] of amorphous phase.

To understand the kinetics of the crystallization process, we tracked the variation of the bonding environment around near-linear chains with the same central atom (Figure S1c). Over the course of crystallization, an octahedral motif with alternating long and short bonds gradually formed, regarded as the genesis of the phase change material[3]. As the crystallization was completed, due to random distribution of anions/cations, the resulting octahedral motif still have a high similarity to the initial amor-state, whereas homopolar and heteropolar bonds were both present. By using a larger supercell (200 atoms, a=16.48 Å, b=20.04 Å, c=19.92 Å) to repeat the simulation crystallization process, similar crystalline atomic model was also obtained as shown in Figure S3. More sepecially, the Sb and Te atoms are random distributed in the cubic lattice, except an abnormal Sb atom locating in the center of the cubic lattice. We also noticed that Konstantinou et al. used a machine-learned, linear-scaling, DFT-accurate interatomic potential to simulate amorphous models of Sb_2Te_3, whose simulation speed is faster than DFT-MD method, this provides an alternative approach to treat larger samples with fewer demands on computing resources[4].

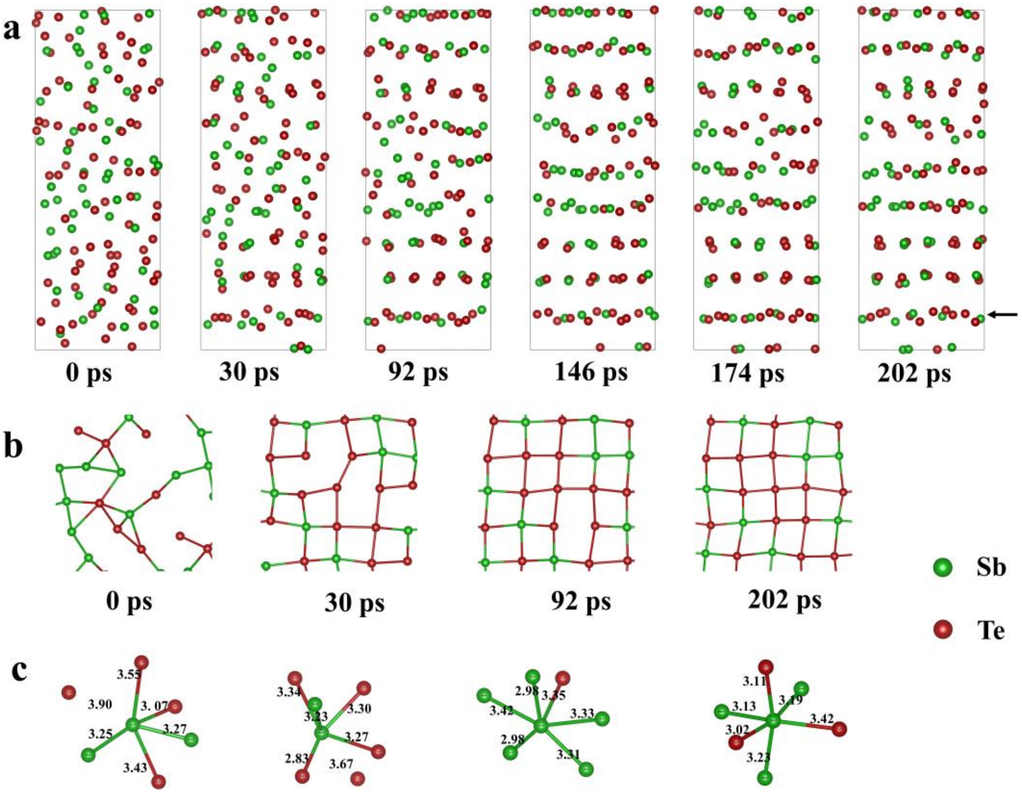

Figure S1 (a) The development of the atomic distribution of the annealed structure at various periods of time at 850 K from amorphous Sb_2Te_3. (b) The atomic arrangement in a single layer at various periods of time. The two-dimensional square lattice structure began to form at times as brief as 30 ps, with the lattice sites randomly occupied by Sb and Te atoms. (c) The variation of the bonding environment around the near-linear chains during crystallization. The entire crystallization process was approximately tens of picoseconds, which completed the atomic ordering process with compositional disorder.

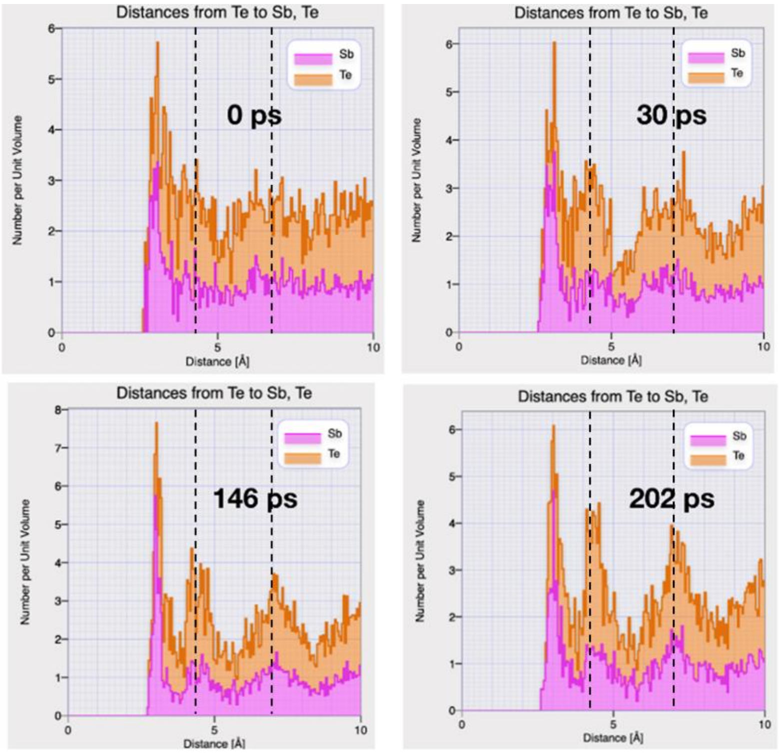

Figure S2. The radial distribution functions of amorphous Sb_2Te_3 annealed at various periods of time at 850 K.

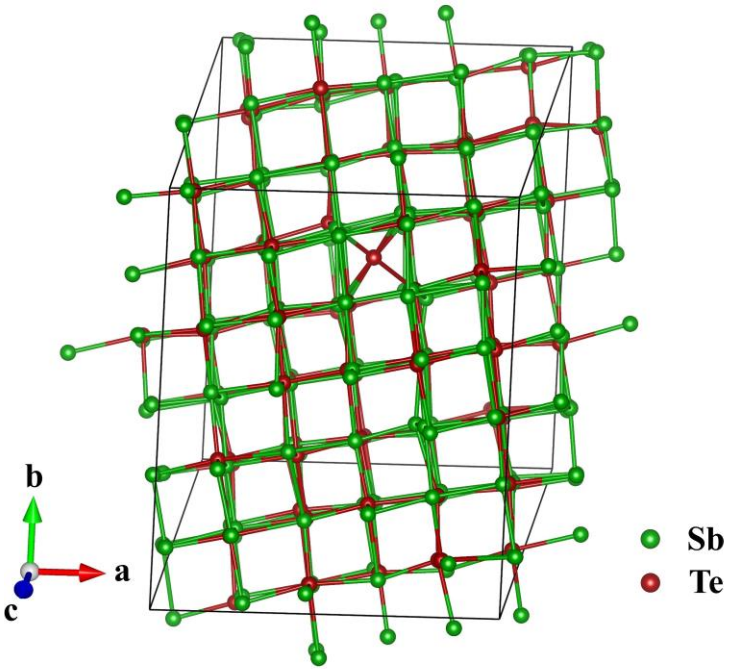

Figure S3. The crystallized atomic model of Sb_2Te_3 alloy with a large supercell (200 atoms).

In order to compare the image difference between sc- and fcc-phase under transmission electron microscope (TEM), we first built the atomic model of the two phases. Afterwards, Crystalmaker and qstem software were utilized to simulate the high angle annular dark field (HAADF) image and fast-Fourier transform (FFT) image inside TEM, respectively. The comparision between the simulated and practical TEM images are depicted in Figure S4-S5. The instrument parameters used in the simulation are the same as those described in the main manuscript. The thickness of the film was set to 15 nm, and the projected direction is along the [110] direction. This is because the image projections of anions and cations atoms in the cubic phase are orderly separated in this direction, as shown in Figure S4, so it can be used as the optimum view direction to directly clarify the atomic arrangement of the cubic phase.

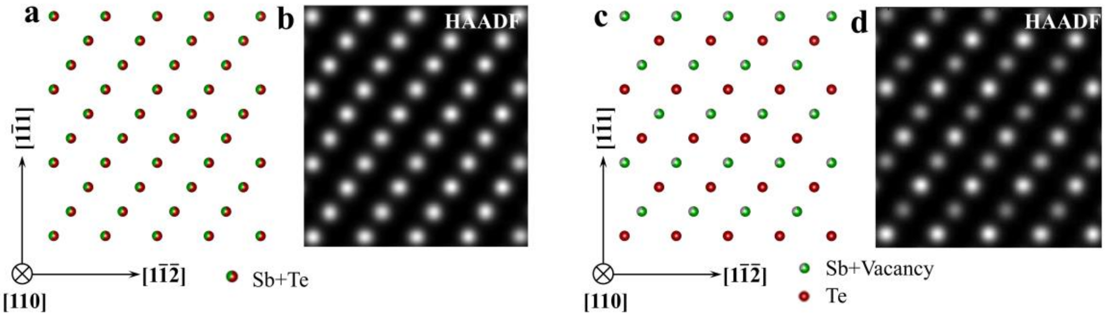

Figure S4. Schematic illustration of (a) atomic model and (b) simulated high angle annular dark field (HAADF) image project along [110] orientation in sc-phase. Schematic illustration of (c) atomic model and (d) simulated HAADF image project along [110] orientation in fcc-phase. It can be seen that the simulated HAADF image in the sc-phase shows uniform contrast, while in the fcc-phase shows periodicity variation contrast as observed in Figure 1c and 1h. This is because the Sb and Te atoms are randomly distributed in sc-phase, while they are ordered distributed in fcc-phase, and the cationic site contains a certain vacancy.

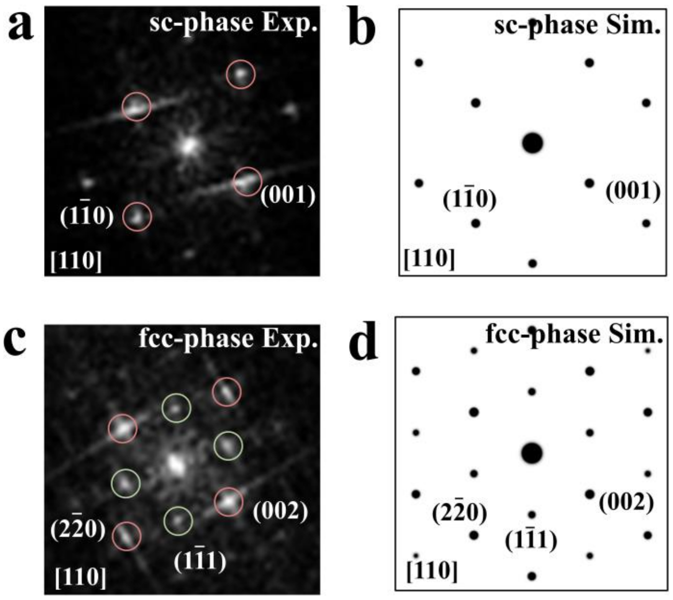

Figure S5. (a) Experimental fast Fourier transform (FFT) patterns and (b) simulated diffraction patterns of sc-phase. (c) Experimental FFT patterns and (d) simulated diffraction patterns of face-centered cubic (fcc-) phases. It can be found that the distribution of diffraction spots in reciprocal space for the simulated and practical images are in good agreement with each other. The distribution of the main spots (pink circles) in FFT patterns from the two phases was nearly the same as discussed in Figure 1d and 1i. However, the fcc-phase exhibited additional (111) diffraction spots (green circles).

Through *in-situ* heating experiments, we further found that the sc-phase will transform into fcc-phase as shown in Figure S6; we also observed an intermediate crystalline grain containing two phases as shown in Figure S7.

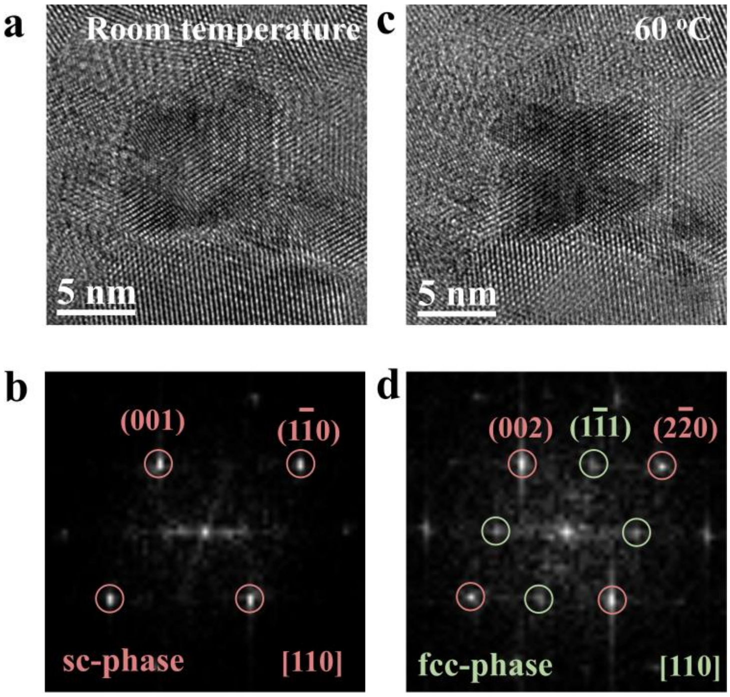

Figure S6. (a) High resolution electron microscopy (HREM) image and (b) the corresponding FFT pattern taken from sc-phase nanograin at room temperature projected along [110] orientation. Increasing the temperature to 60 °C, (c) HREM image and (d) the corresponding FFT pattern demonstrate the phase transition from sc- to fcc-phase occurred in the inspected nanograin.

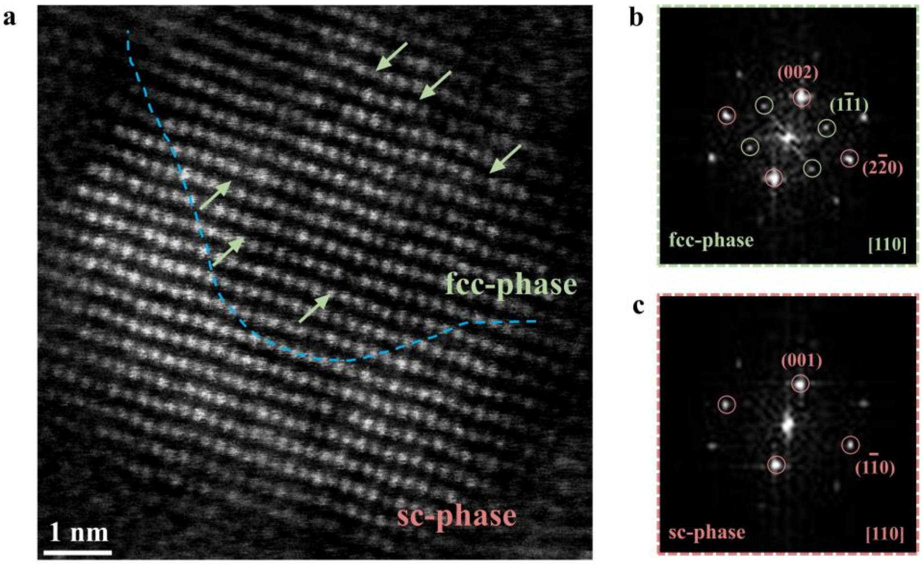

Figure S7. Atomic arrangement in a two phase co-existed grain. (a) The HAADF image, where the upper right area is fcc-phase and the bottom left area is sc-phase, viewed along [110] direction. The dashed blue line is the phase boundary. Light green arrows denote the dark

atomic columns, indicating that vacancies have been formed at these layers. (b) and (c) are the corresponding FFT patterns taken from fcc- and sc-areas, respectively.

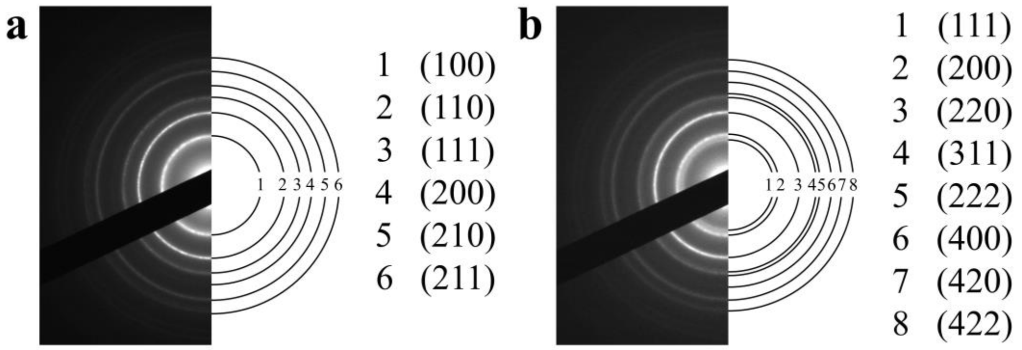

Figure S8. The SAED patterns of as-deposited Sb_2Te_3 films by using different prepared ways: (a) co-sputtering Sb and Te targets, (b) sputtering Sb_2Te_3 single targe. A more disordered structure can be obtained by means of co-sputtering. Similar to Figure S4, the two cubic structures have common main diffraction rings in SAED patterns, while the fcc-phase has additional diffraction rings.

Table S1 Plane indices of the simple cubic (sc-) phase of the as-deposited Sb_2Te_3 film by co-sputtering Sb and Te targets. The plane index of the sc-phase based on the selected area electron diffraction (SAED) patterns, where R is the radius of diffraction rings in reciprocal space, and D [experiment] is the plane spacing obtained from the measured data, while D [theory] is the computational data using sc-lattice constant a = 3.05 Å. The consistence between both D values is pretty good.

	R (1/nm)	R^2 (1/nm^2)	R^2 ratio	D [experiment] (nm)	D [theory] (nm)	Lattice Plane
1	3.279	10.751	1	0.305	0.305	(100)
2	4.820	23.236	2.161	0.207	0.207	(110)
3	5.829	33.984	3.161	0.172	0.176	(111)
4	6.524	42.568	3.959	0.153	0.153	(200)
5	7.592	57.645	5.362	0.132	0.135	(210)

6	8.084	65.351	6.07	0.124	0.125	(211)

Table S2 Plane indices of the fcc-phase of Sb_2Te_3 film by sputtering Sb_2Te_3 single target. The plane index of the fcc-phase based on the SAED patterns, where R is the radius of diffraction rings in reciprocal space, and D [experiment] is the plane spacing obtained from the measured data, while D [theory] is the computational data using fcc-lattice constant a = 6.10 Å. The consistence between both D values is pretty good.

	R (1/nm)	R^2 ($1/nm^2$)	R^2 ratio	D [experiment] (nm)	D [theory] (nm)	Lattice Plane
1	2.844	8.088	3	0.352	0.352	(111)
2	3.224	10.394	3.855	0.310	0.305	(200)
3	4.629	22.024	7.948	0.216	0.216	(220)
4	5.470	29.921	11.098	0.183	0.184	(311)
5	5.690	32.376	12.001	0.176	0.176	(222)
6	6.595	44.342	15.916	0.152	0.153	(400)

7	7.435	55.279	19.842	0.135	0.136	(420)
8	8.001	64.016	23.745	0.125	0.125	(422)

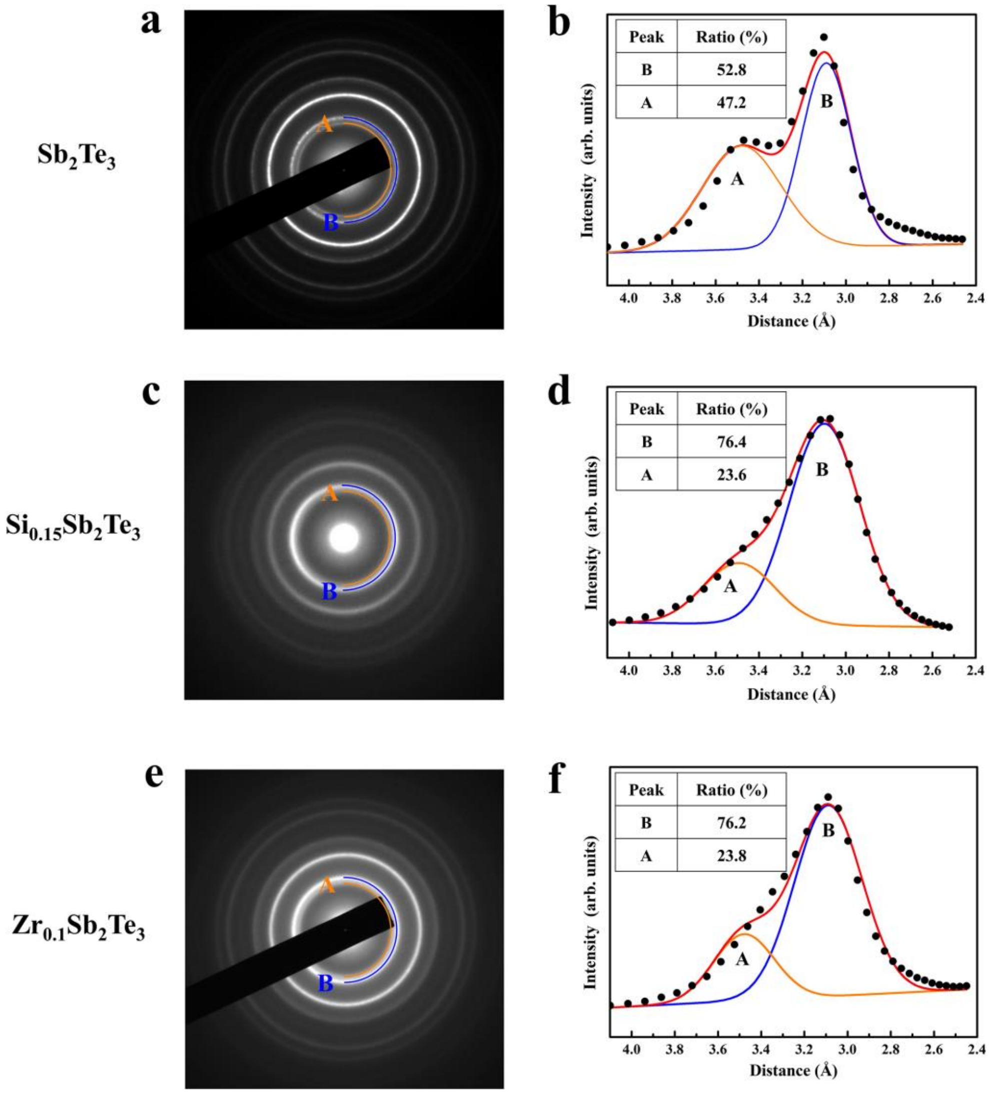

Figure S9. Microstructure characterization in Sb_2Te_3, Si doped Sb_2Te_3 and Zr doped Sb_2Te_3 films. (a) The polycrystalline diffraction rings of (a) as-deposited Sb_2Te_3, (b) crystallized $Si_{0.15}Sb_2Te_3$ (annealed at 140°C) and (c) crystallized $Zr_{0.1}Sb_2Te_3$ (annealed at 140°C) films. The distance of orange semi-circular ring is ~3.49 Å, which only belongs the {111} in fcc-phase, and is defined as peak A. The distance of blue semi-circular ring is ~3.09 Å, which belongs {200} lattice plane in fcc-phase or {100} lattice plane in sc-phase, and is defined as peak B. The raw radially integrated diffraction curves (b), (d) and (f) of electronic diffraction intensity extracted from the SAED patterns of Sb_2Te_3, $Si_{0.15}Sb_2Te_3$ and $Zr_{0.1}Sb_2Te_3$ respectively. By comparing the area integral ratio variation between peak A and peak B, the former gets smaller after elemental doping, demonstrating that the doped Sb_2Te_3 films retain more sc-phase. Above results shows that doping with additional chemical elements can improve the thermal stability

of Sb-Te material, Si or Zr doping element can stabilize the sc-phase of Sb_2Te_3 material to 140°C.

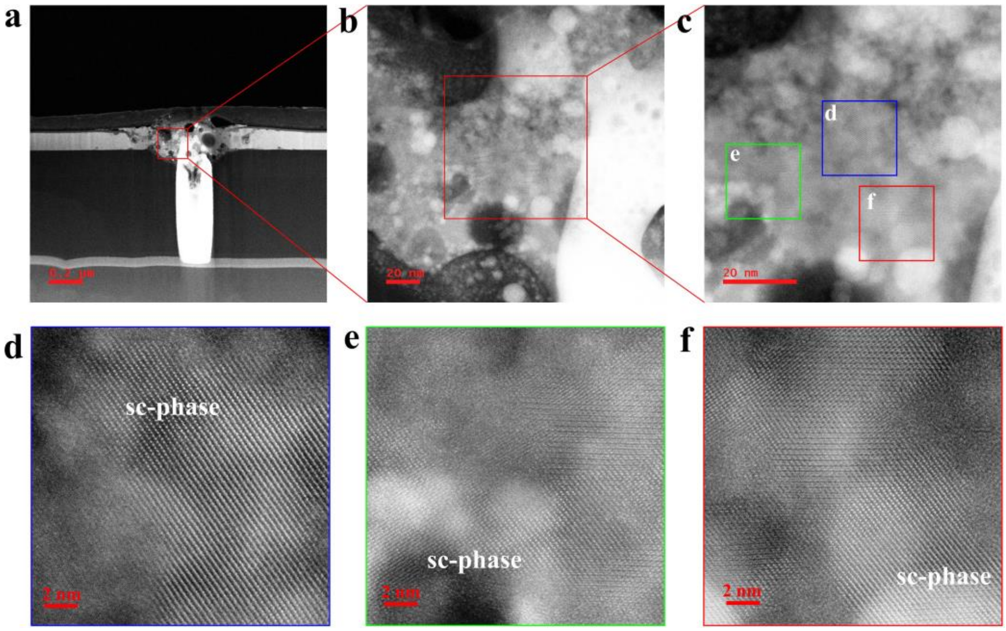

Figure S10. (a) Cross-sectional HAADF image of PCM device based on doped-Sb_2Te_3 material at SET state. (b-c) Enlarged HAADF images shows the investigated crystalline areas. (d-f) Magnified HAADF images taken from the blue, green and red box in c, respectively. In each image, sc-phase with uniform contrast can be observed.

When projected along [110] orientation, HAADF image shows uniform contrast in nanocrystalline at the initial stage of crystallization of SbTe alloy (Sb:Te=1:1) as shown in Figure S11a. Similar to other components of Sb-Te alloys, atomic EDS mappings of elements Sb and Te as well as the overlap, respectively, show random distribution of Sb and Te atom in Figure S11b-d, which fits well with the sc-phas.

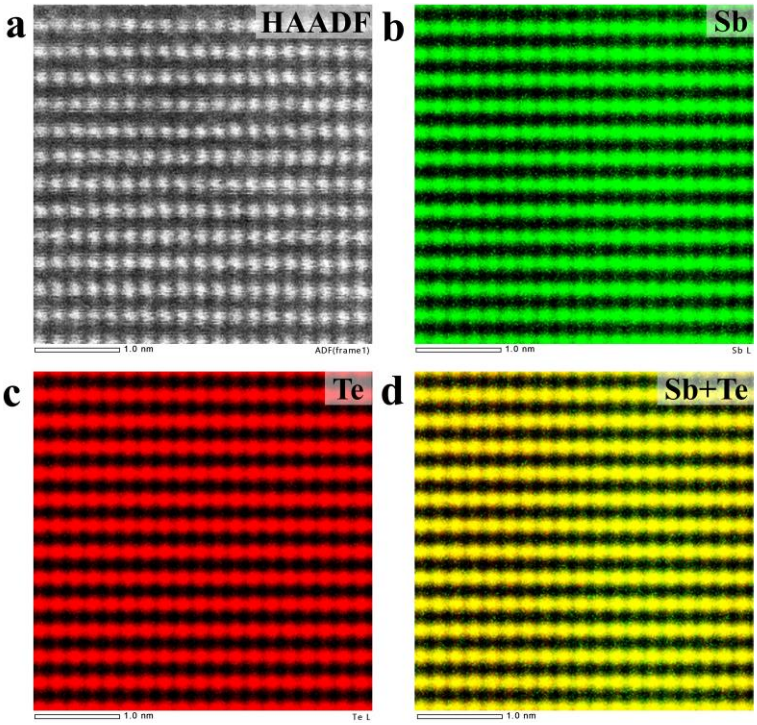

Figure S11. HAADF images of sc-phase projected along the [110] orientation in SbTe alloy. (b–d) Atomic EDS mappings of elements Sb and Te as well as the overlap, respectively.

With the increasing of temperature, the COP will continue to proceed in SbTe alloy. Figure S12 is a picture of a typical layer ordered hex-phase structure at a higher temperature, where obvious van der Waals gaps can be seen due to the directed migration of Sb and Te atoms.

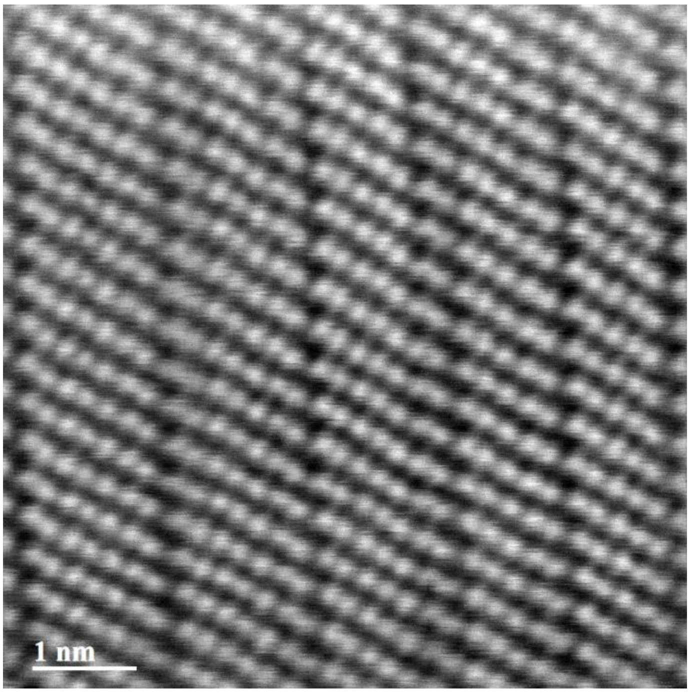

Figure S12. HAADF images of hex-phase projected along the $[2\bar{1}\bar{1}0]$ orientation in SbTe alloy.

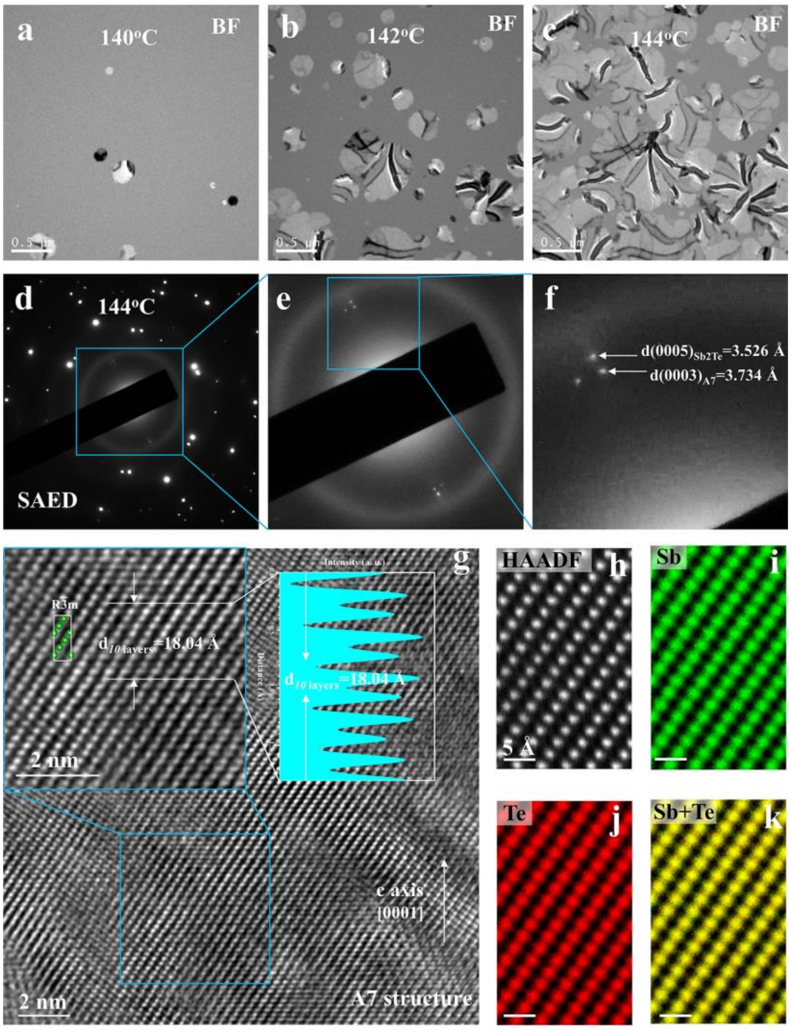

Figure S13. (a-c) The Sb_2Te film was heated in TEM by an in-situ heating holder, and stopped at 144°C. From the corresponding (d) SAED pattern, it can be seen that there still has (e) amorphous ring with diffraction spots together. As can be seen from the enlarged SAED pattern in panel f, except for the diffraction spot with d = 3.526 Å belonging to (0005) lattice plane of hex-phase, there is another spot with d = 3.734 Å belonging to A7-(0003). (g) HREM image of the initial crystallization structure with A7 structure. Though bilayer can be detected, the quintuple layer is not obvious. The layered structure with alternative quintuple layer and bilayer hasn't formed at this stage. (h-k) The atomic EDS mappings of elements Sb, Te and the overlap respectively, taken from the initial crystallized Sb_2Te film, showing the mix of elements.

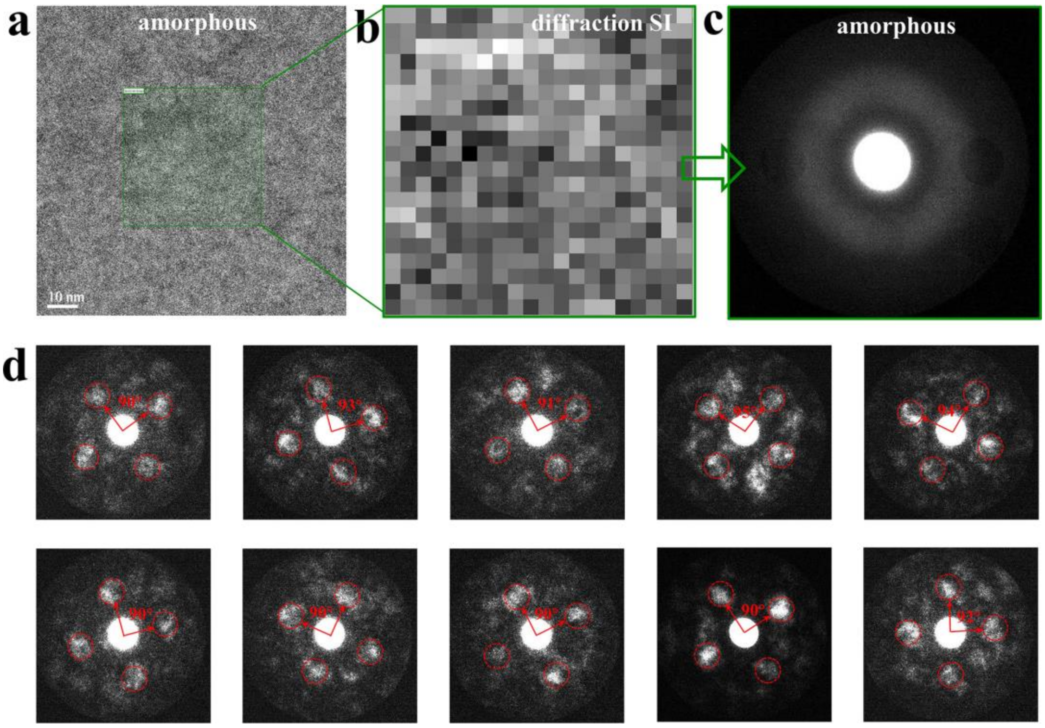

Figure S14. (a) Scanning transmission electron microscopy (STEM) image of the amorphous Sb_2Te film, which shows a uniform contrast. (b) Diffraction SI image taken from the green square in panel a with the help of the automatic scanning function of the STEM system to investigate the local structure of amorphous film, in which each pixel contains an angstrom-beam electron diffraction (ABED) pattern. To obtain the angstrom parallel electron beam, the condenser lens aperture with a radius of 5 um was superimposed, the convergence angel is ~2 mrad, and the beam size is ~7.5 Å. (c) The sum of ABED patterns (20pixel*20pixel) in panel b, which shows diffused amorphous pattern. (d) Representative ABED patterns taken from a single pixel in panel b. Note that the ABED patterns closely resemble the typical crystalline patterns with four-fold symmetry (relative angle is ~90°), suggesting the existing of distorted octahedral configuration in the amorphous state[5].

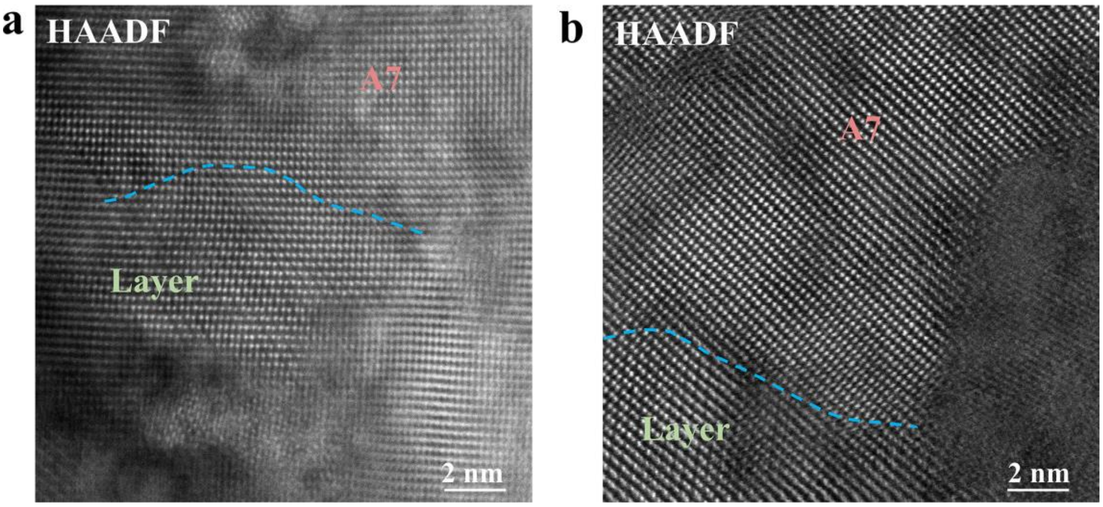

Figure S15. HAADF images of crystallized $Ta_{0.1}Sb_2Te$ film (a: annealed at 220°C) and crystallized $Cr_{0.4}Sb_2Te$ film (b: annealed at 250°C) projected along $[1\bar{1}00]$ orientation, respectively, showing the transition snapshot from A7 to layered structure. Above results show that Ta or Cr element can stabilize the A7 phase of Sb_2Te material to 220°C and 250°C, respectively

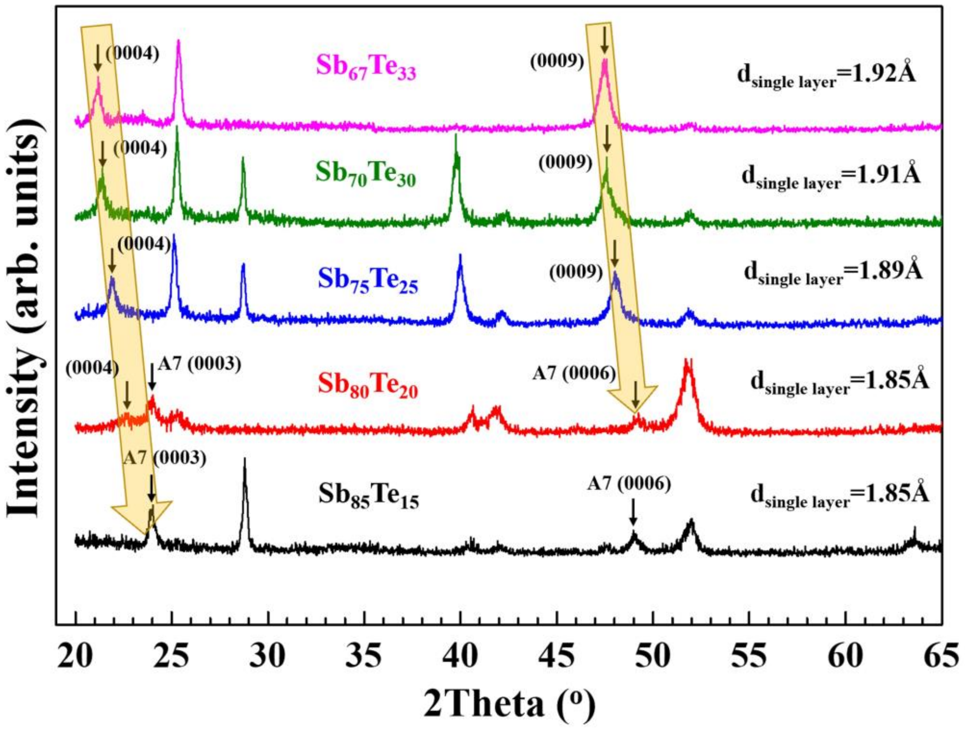

Figure S16. XRD patterns obtained from different composition of Sb-Te films annealed at 240 °C. A clear shift in peak position can be seen, especially on peak (0004) and peak (0009). With the increase of Sb content, the structure becomes more and more like A7 at the same thermal treatment condition. Single layer spacing was obtained from the peak (0009).

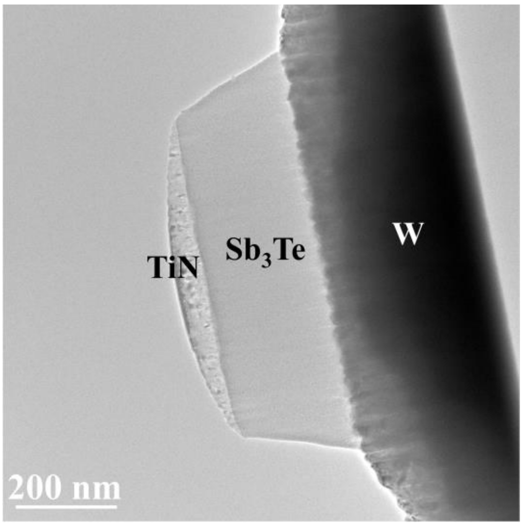

Figure S17. TEM image of a prototype W/Sb_3Te/TiN nano-pillar device prior to electrical operation.

Movie S1. *In situ* HREM video records the formation of layered structure from A7 phase under external electric field. The observing area is ~50 × 50 nm^2. In this movie, the formation of layered structure can be clearly observed, accompanied by the decreasing of resistance.

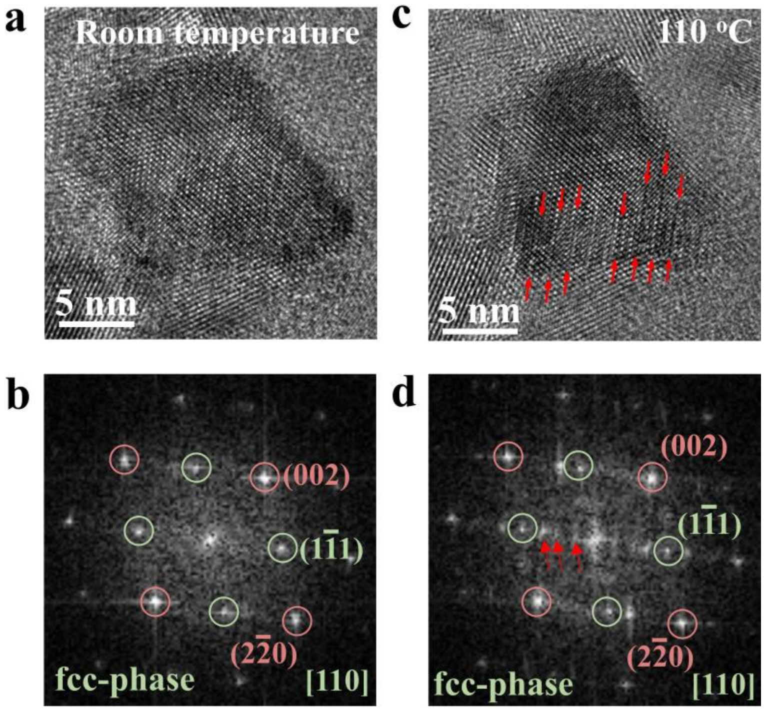

Figure S18. (a) HREM image and (b) the corresponding FFT pattern taken from a fcc-phase nanograin at room temperature projected along [110] orientation. Increasing the temperature to 110 ^{o}C, vacancy ordering layers are observed in HREM image (c) denoted by red arrows, and additional modulated spots also appear in its corresponding FFT pattern (d), ascribing to the vacancy ordering in the inspected nanograin.

Coordinates of Sb_2Te_3 systems

```
amor-23-state I
1.0
      10.6192998886         0.0000000000         0.0000000000
       0.8507370764         7.3719740421         0.0000000000
       1.9823759395        -1.5629300348         6.2554422479
   Sb   Te
    6    9
Cartesian
     2.242429880         2.510696236         4.492408240
     9.303683921         4.428832510         3.523065055
    10.090711816         1.642829870         3.934610585
     5.942937443         5.263200773         1.598390517
     3.822814480         5.593612061         5.673686152
     6.939881411         4.742036815         5.278467127
     3.262256146         0.076814006         3.112833087
     3.282158752         3.617797665         1.626039648
    11.390759128         5.375832612         5.400510997
     1.624056423         5.915095589         2.142801801
    10.981675744         2.216120284         0.730135209
     7.232657887        -0.040594698         6.053829222
     5.707223291         1.519447880         4.040140032
     7.973473293         3.435410540         0.656508685
     7.203065646         0.175546157         1.779986161

Sb2Te3-sc-state II
1.0
      11.5369033813         0.0000000000         0.0000000000
       0.8299093285         4.2547776325         0.0000000000
       0.8160038832        -2.3465893578         9.7358347761
   Sb   Te
    6    9
Cartesian
     5.934225122         2.356120349         6.525532031
     1.045552000         3.354538690         2.454233249
     1.744226656         1.003359922         0.756841804
     8.415970229         1.894334007         2.667419579
     1.828448014         0.979200714         6.494441246
     9.209045948        -0.403856676         6.444771544
```

3.484517643 2.902749955 4.553627541
6.801957099 0.005559906 4.632368871
4.198160206 0.603053249 2.464475852
10.842298793 1.461845388 4.655684783
4.269478594 0.489027933 8.208176215
11.766023203 -0.906913351 8.223156685
5.971135548 2.403554495 0.831817197
8.359875301 1.933849997 8.279256121
10.068724611 3.790651581 0.958807288

Sb2Te3-fcc-state III
1.0
11.4003753662 0.0000000000 0.0000000000
-0.0723878663 10.5117479808 0.0000000000
-0.8077408645 0.0400758807 4.2228813826
Sb Te
6 9
Cartesian
-0.195403287 4.264564133 2.123856462
9.517030327 8.676110368 3.897403536
2.375987783 8.055651017 2.566909724
7.353552355 4.635132686 3.516489286
3.881347402 4.518552762 0.759808203
5.047715137 0.829231003 3.058328790
4.723126405 6.388368817 3.071286226
6.713113268 9.398945974 4.139349097
1.365675842 6.288403406 0.209533061
-0.138946020 10.093545555 2.034786451
6.415129285 2.762336927 1.163996580
9.489578892 2.652925590 4.003809363
3.595727237 10.025978669 0.613441135
8.831996668 6.354568251 1.619409231
2.234131407 2.722330433 2.647640048

Sb2Te3-fcc-vacancy ordering state IV
1.0
11.3106002808 0.0000000000 0.0000000000
-0.0722850634 10.5410519761 0.0000000000
-0.7780249157 0.0155991891 4.2015426779
Sb Te
6 9
Cartesian
6.252087564 8.279164830 1.280840336
-0.179073798 4.364926747 2.096317666
9.541045902 8.346266613 3.965920233
2.249316906 8.336205997 2.533824430
7.085887912 4.372954828 3.577151438
3.815794288 4.301935633 0.830644962
4.623990177 6.315438728 3.145568881
7.070835224 10.016215361 3.606436142
1.435713294 6.331782774 0.222723785

-0.173949594	10.064715213	2.076234315
6.278012221	2.631006806	1.266933126
9.520627326	2.655579957	3.967474661
3.872450236	10.027925701	0.684599345
8.710393559	6.345709112	1.674062679
2.196064336	2.604432997	2.683693383

Sb2Te3 hex-phase state V
1.0

4.3193802834	0.0000000000	0.0000000000
-2.1582006413	3.7415526221	0.0000000000
0.0311194512	-0.0179843988	30.2959534108

Sb Te
6 9
Cartesian

-2.145584806	3.734237298	12.062817708
2.183208278	1.233858618	22.161474564
0.003265703	2.493031715	1.964115483
4.337871547	-0.010707861	18.233114936
2.189026546	1.230495700	28.331832285
0.009077650	2.489672597	8.134458984
-2.133434376	3.727219988	23.864046581
2.164188909	1.244847663	3.666580056
0.015363995	2.486040294	13.765311174
4.325720757	-0.003689485	6.431913150
2.176923582	1.237490117	16.530662100
0.028107390	2.478676446	26.629301800
2.192312196	3.723537456	30.295938965
2.170542749	1.241176202	10.098606293
0.021745252	2.482354335	20.197340797

Coordinates of Sb_2Te systems

amor-21-state I
1.0

6.8488001823	0.0000000000	0.0000000000
1.2283406472	6.3686243449	0.0000000000
0.4929175774	0.8391371977	6.4601060305

Sb Te
6 3
Cartesian

1.255133282	2.204493089	5.731922797
3.101783177	5.898630438	4.173422356
7.294211801	5.141951199	5.500457448
2.443417034	3.169149679	3.153500762
3.219742288	0.740089052	0.958938161
6.629783995	4.336915735	2.771191743
5.150968411	1.749890933	4.856190978
6.430147995	0.638698460	2.113552983
3.913528278	4.243394741	0.245484021

Sb2Te-state II
1.0

9.4296360016	0.0000000000	0.0000000000
-0.0126684170	4.3360796880	0.0000000000
-2.5670358695	2.1594479833	6.8576602071

Sb Te
6 3
Cartesian

7.221607560	3.375190052	2.911948540
0.780591225	1.204180714	0.719580918
7.130519179	1.202648985	0.935088505
0.921840925	3.360265329	3.122911560
4.070913040	3.375345802	3.038477425
1.132360536	5.536335051	5.172540562
3.954081119	1.200207818	0.824666197
-1.946377623	5.518812546	5.537520184
4.121806091	5.545561607	5.168205785

Sb2Te-state III
1.0

4.3575267792	0.0000000000	0.0000000000
-2.1772587210	3.7755682205	0.0000000000
-0.0029826247	0.0012398950	17.2699543793

Sb Te
6 3
Cartesian

2.421751736	1.680825207	11.288535286
2.418500796	1.678056125	5.980980067
0.240527999	2.935298114	7.514227990
0.240777470	0.421162118	15.320310025
0.241580383	2.934298685	1.950210379
0.242221239	0.423693229	9.755193691
2.421520350	1.677443448	0.000318017
0.241721515	2.939722656	13.626556634
0.240767040	0.416590749	3.643244112

References

[1] S. Caravati, M. Bernasconi, M. Parrinello, *Phys. Rev. B* **2010**, *81*, 014201.

[2] F. C. Mocanu, K. Konstantinou, J. Mavračić, S. R. Elliott, *Phys. Status Solidi RRL* **2021**, *15*, 2000485.

[3] Z. T. Song, R. B. Wang, Y. Xue, S. N. Song, *Nano Res.* **2021**, *15*, 765.

[4] K. Konstantinou, J. Mavračić, F. C. Mocanu, S. R. Elliott, *Phys. Status Solidi B,* **2021**, *258*: 200046

[5] A. Hirata, T. Ichitsubo, P. F. Guan, T. Fujita, M. W. Chen, *Phys. Rev. Lett.* **2018**, *120*, 205502.
